# Possible Relation between Lack of Posterior Vitreous Detachment and Severe Endogenous Endophthalmitis

**DOI:** 10.1155/2016/8561379

**Published:** 2016-09-27

**Authors:** Kazuhiko Umazume, Jun Suzuki, Yoshihiko Usui, Yoshihiro Wakabayashi, Hiroshi Goto

**Affiliations:** Department of Ophthalmology and Visual Sciences, Tokyo Medical University, Tokyo, Japan

## Abstract

*Purpose*. Endogenous endophthalmitis (EE) is a rare ocular disease caused by bacterial or fungal infection of intraocular spaces by hematogenous spread of pathogens from distant infectious loci in the body. We investigated the clinical characteristics and management of eyes with EE in ten consecutive patients.* Methods*. Ten patients (10 eyes) with EE treated at Tokyo Medical University Hospital in 2014 were reviewed. We retrospectively studied the causative organisms, systemic complications, pre/postoperative mean best-corrected visual acuity (BCVA), and status of posterior vitreous detachment (PVD).* Results*. The 10 patients comprised 8 males and 2 females, with mean age of 71.2 years. The causative organisms were bacteria in 6 eyes and fungi in 4 eyes. Systemic complications included septicemia or disseminated intravascular coagulation in 5 patients and diabetes mellitus in 4 patients. Postoperative BCVA was improved by 0.2log⁡MAR or greater in 4 eyes and decreased in 4 eyes. Vitrectomy was performed in all eyes, and 4 required multiple surgeries. During vitrectomy, PVD was absent in 8 eyes, 4 of which showed retinal necrosis. The mean age of patients with no PVD was 71.2 years.* Conclusion*. Despite an advanced age, PVD was absent in the majority of patients with EE. PVD may be related to the pathogenesis and aggravation of EE.

## 1. Introduction

Metastatic endogenous endophthalmitis is a potentially sight-threatening intraocular infection resulting from hematogenous spread of microorganisms from a distant infective source within the body. The incidence of endogenous endophthalmitis is far lower than that of exogenous endophthalmitis including postoperative endophthalmitis, accounting for only 2 to 8% of all endophthalmitis cases [1, 2]. The causative microorganisms are divided into bacteria and fungi, most commonly* Candida* species. Among bacteria, Gram-positive bacteria are common in developing countries, while Gram-negative bacteria have been reported in East Asia region [3–5]. Compared to fungal infections, bacterial infections spread rapidly to intraocular tissues and require urgent management.

Basic local treatments for endogenous endophthalmitis include intravitreal injection of antibacterial or antifungal agents against the causative microorganisms as well as vitrectomy. In the case of phakic eyes, lens extraction is conducted simultaneously, and in the case of eyes with implanted intraocular lens, extraction of the intraocular lens should be considered as the situation demands. In addition, frequent instillation of ceftazidime and vancomycin eye drops after surgery is an accepted treatment for bacterial endophthalmitis [6, 7] and should be started as soon as possible after operation.

The predisposing risk factors for metastatic endogenous endophthalmitis include urinary tract infection, diabetes, HIV infection, intravenous hyperalimentation, liver abscess, and infectious endocarditis, while long-term hospitalization that has increased with the progression of population aging is also an important risk factor [5, 6, 8, 9]. Furthermore, the association of posterior vitreous detachment (PVD) with retinal disease has attracted attention, accompanying the advent of optical coherence tomography [10, 11]. The relationship between PVD and the pathogenesis or severity of endogenous endophthalmitis has not been reported. In the present study, we examined PVD pre- and intraoperatively in patients with endogenous endophthalmitis and correlated the presence of absence of PVD with clinical features.

Recently, the aging society has become an issue mainly in industrial countries, and the increase of endogenous endophthalmitis accompanying aging has been a concern. In this report, we describe the clinical characteristics, surgical methods, and outcomes of 10 cases of endogenous endophthalmitis treated in our department during the previous one year.

## 2. Methods

Ten consecutive patients (10 eyes; 4 left eyes and 6 right eyes) diagnosed with metastatic endogenous endophthalmitis at the Tokyo Medical University Hospital between April 2014 and March 2015 were studied. The patients comprised 8 males and 2 females, with mean age of 71.2 ± 10.5 years. All patients underwent 25-gauge vitrectomy using the Constellation Vision System (Alcon Laboratories Inc., Fort Worth). Ceftazidime and vancomycin were added to Balanced Salt Solution (BSS) to obtain final concentrations of 20 mg/mL and 1 mg/mL, respectively, and used as intraocular irrigation solution. During surgery, vitreous fluid sample was collected for culture, smear, and microscopic examination to identify the causative microorganism. Blood culture was also performed before surgery.

The parameters analyzed were causative microorganism, clinical findings, pre- and postoperative visual acuity, surgical method, systemic disease, and PVD. Clinical findings focused on chemosis, keratic precipitates, fibrin deposition in anterior chamber, hypopyon, and fundus visibility. Posterior vitreous detachment was assessed by B mode ultrasonography before surgery and by microscopic observation during surgery.

Preoperative and postoperative visual acuity were compared by Student's *t*-test. Statistical analyses were performed using MedCalc version 12.1.1 (Mariakerke, Belgium). *P* values less than 0.05 were considered significantly different.

## 3. Results

The causative microorganisms isolated from vitreous fluid samples collected during surgery and from blood cultures were bacteria in 6 eyes and fungi in 4 eyes. The bacterial species isolated were* Klebsiella* species in 2 eyes,* Escherichia coli* in 1 eye,* Nocardia* species in 1 eye, and unidentified Gram-negative bacteria in 2 eyes. The fungal species isolated were* Candida* species in 3 eyes and* Cryptococcus* in 1 eye (Table 1).

Preoperative clinical findings in all 10 eyes (Table 2) included chemosis in 7 eyes, keratic precipitates in 9 eyes, fibrin deposition in 8 eyes, hypopyon in 6 eyes, and invisible fundus in 5 eyes. Among the 4 eyes with fungal endophthalmitis, the fundus was visible in 3 eyes and difficult to observe in only 1 eye. Hence, fungal endophthalmitis appears to show slower progression of inflammation clinically compared to bacterial endophthalmitis.

**Figure 1:**
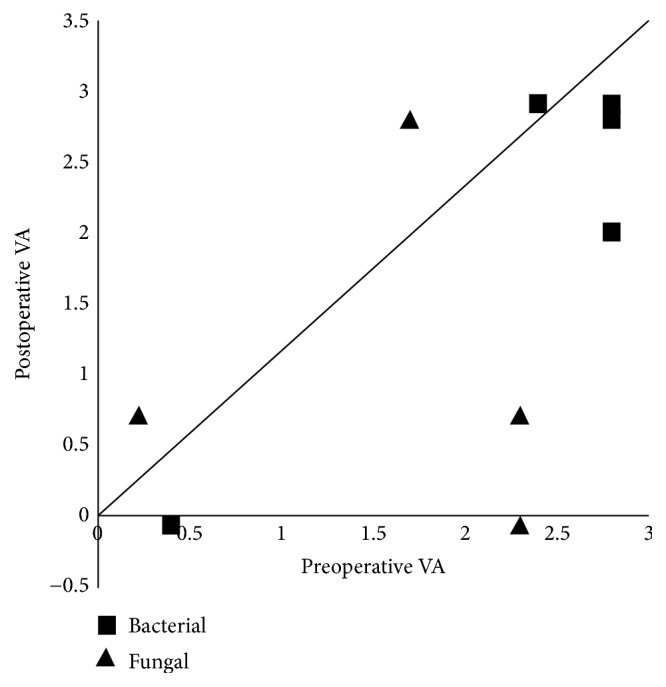
Comparison of pre- and postoperative visual acuity in 10 eyes treated for endogenous endophthalmitis. VA: visual acuity.

When improvement in visual acuity was defined as gain of more than 0.2log⁡MAR, postoperative visual acuity was improved in 4 eyes, unchanged in 2 eyes, and deteriorated in 4 eyes (Figure 1). In this analysis, the logMAR equivalent for counting fingers was 1.85, hand motion was 2.3, light perception was 2.8, and no light perception was 2.9 [12]. Four eyes (40%) were able to avoid becoming socially blind (>0.7 log MAR). Comparison of final visual acuity between bacterial endophthalmitis and fungal endophthalmitis showed that visual acuity tended to be slightly better in fungal endophthalmitis (1.03 ± 1.24) compared to bacterial endophthalmitis (2.24 ± 1.18), although there was no significant difference between two groups. Three eyes lost light perception after surgery. All three had bacterial endophthalmitis, caused by* Klebsiella* species,* Escherichia coli*, and* Nocardia* species in 1 eye each.

The risk factors in the 10 patients in the present study were diabetes in 4 patients, anastomotic leakage after colon cancer surgery in 2 patients, ruptured liver abscess in 1 patient, aspiration pneumonia in 1 patient, and long-term oral steroid therapy in 1 patient. Serious systemic complications consisted of septicemia in 1 patient and disseminated intravascular coagulation in 4 patients.

Regarding treatment method, all 10 eyes underwent vitrectomy, and 4 (40%) required multiple surgeries before inflammation was resolved. In only one eye with severe pain and loss of light perception, eyeball enucleation was eventually conducted. In 2 eyes that had been implanted with intraocular lens before surgery, the intraocular lenses were extracted in both eyes.

**Figure 2:**
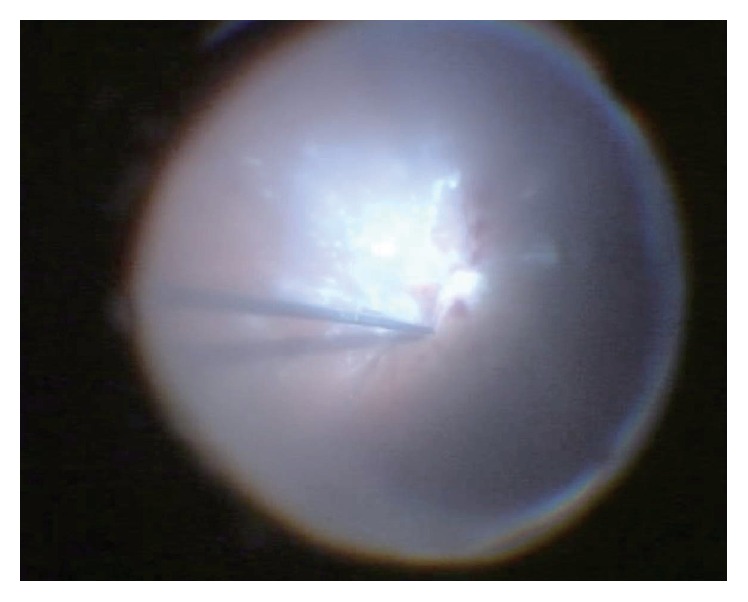
Fundus image shows induction of posterior vitreous detachment.

Posterior vitreous detachment was absent in 8 eyes (80%) before surgery. In these eyes, PVD was induced intentionally during surgery. In this series, a high frequency of the absence of PVD was observed (Figure 2).

## 4. Discussion

According to an epidemiological survey of intraocular inflammations in Japan conducted in 2007, endogenous endophthalmitis accounted for 1 to 2% of intraocular inflammations [13]. Despite being a relatively rare intraocular disease, we observe a trend of increase at the Tokyo Medical University Hospital. While we treated a total of 13 cases between 2006 and 2013, we encountered 10 cases during one year in 2014. Regarding the age of disease onset, the patients with acute anterior uveitis (m = 147), which is differential diagnosis of endogenous endophthalmitis, were 43.2 ± 14.8 years old. On the other hands, the patient with endogenous endophthalmitis in this series was 71.2 ± 10.5 years old. With the progression of population aging, the increase in endogenous endophthalmitis in the future is a concern.

The clinical picture of endogenous endophthalmitis is diverse. The misdiagnosis rates at presentation ranged from 16 to 63% [3, 14]. The early clinical symptoms include pain, hyperemia, floaters, and decreased vision. However, since some patients are not capable of complaining about the symptoms due to poor general condition, many patients are diagnosed in a serious state. In the present series, high rates of severe inflammatory findings were observed: fibrin deposition in 8 patients (80%) and hypopyon in 6 patients (60%). Furthermore, many patients with bacterial endophthalmitis had invisible fundus, probably as a result of rapid exacerbation of inflammation.

In the present study, all 10 eyes underwent vitrectomy. When inflammation spreads to the vitreous, prompt dissection of the vitreous is necessary. Although whether the causative organisms are bacteria or fungi can only be identified after performing culture of vitreous samples, we conducted surgery using an intraocular irrigation solution containing a mixture of vancomycin (1 mg/mL) and ceftazidime (20 mg/mL), as reported previously [15]. Although the vitreous body has to be dissected as much as possible, induction of an iatrogenic retinal break should be avoided, because this may affect retinal reattachment. In principle, we replaced the vitreous cavity with the intraocular irrigation solution containing antibiotics, but we used silicon oil when retinal tear was observed during surgery. In 4 patients (40%), the first vitrectomy failed to resolve inflammation and multiple surgeries were required. In one patient with bacterial endophthalmitis, loss of light perception and uncontrolled pain eventually led to enucleation. Patients with endogenous endophthalmitis usually present with pain, and due to the high risk of expulsive hemorrhage during surgery, conducting surgery under general anesthesia is recommended if the general condition of the patient can tolerate anesthesia [16, 17].

The present study also examined the relation between PVD and endogenous endophthalmitis. Posterior vitreous detachment was not found before surgery in 8 eyes (80%), and PVD was induced during surgery in these eyes. The patients with no PVD had a mean age of 73.4 ± 8.4 years, which was older than the mean age of 54 to 57 years reported for the occurrence of PVD [18]. Furthermore, retinal necrosis is known to occur secondary to severe inflammation in endogenous endophthalmitis. In the present study, retinal necrosis was observed in 4 eyes (40%), all of which had no PVD before surgery. These findings suggest that contact between the retina and the vitreous body may be associated with severe disease. A possible reason is that the gel-like vitreous body plays the role as a growth medium for the causative microorganisms.* In vitro* study has shown that the vitreous is associated with cell proliferation [19]; the same phenomenon may occur also* in vivo*. Currently, the use of ocriplasmin for vitreolysis has attracted interest [20, 21], and use of this agent may also prevent aggravation of endogenous endophthalmitis.

Although the causative microorganisms have not changed compared to past reports, endogenous endophthalmitis remains a disease with poor visual outcome despite the advances in vitreous surgery. With the continuous progression of population aging, the number of cases is expected to increase in the future, and appropriate management including early diagnosis is required.

## Figures and Tables

**Table 1:** Cultures results.

Species	Number of eyes
Bacteria	
*Klebsiella pneumonia*	2
*Escherichia coli*	1
*Nocardia*	1
Gram-negative bacteria (detail unknown)	2
Fungus	
*Candia albicans*	3
*Cryptococcus*	1

**Table 2:** Clinical features.

Symptoms	Number of eyes	Number of eyes (fungal)
Chemosis	7	2
Keratic precipitates	9	4
Fibrin	8	3
Hypopyon	6	3
Indistinct of fundus	5	1
